# Correction to “Radiotherapy and Cardiovascular Disease Survival Risk in Non‐Small Cell Lung Cancer: A Population‐Based Study From the Surveillance, Epidemiology, and End Results Database”

**DOI:** 10.1002/cam4.72081

**Published:** 2026-07-03

C. Fan , C. Hao , L. Liu , et al., “Radiotherapy and Cardiovascular Disease Survival Risk in Non‐Small Cell Lung Cancer: A Population‐Based Study from the Surveillance, Epidemiology, and End Results Database,” Cancer Medicine 15, no. 6 (2026): e71965, 10.1002/cam4.71965.42286978 PMC13263541

The author order was incorrect and has been changed from Li Liu, Chunhui Fan, Wenxin Ren, Mingyi Wen, Cunqi Li, Cuiping Hao, Yian Geng, Liping Zhang to Chunhui Fan, Cuiping Hao, Li Liu, Mingyi Wen, Cunqi Li, Yian Geng, Wenxin Ren, Liping Zhang.

The online version of this article has been corrected accordingly.

We apologize for this error.

